# Interlesion Time as a Key Metric of Procedural Proficiency in Atrial Fibrillation Ablation: From Fellow to Attending

**DOI:** 10.1002/joa3.70360

**Published:** 2026-05-14

**Authors:** Hitoshi Mori, Daisuke Kawano, Masataka Narita, Kazuhisa Matsumoto, Tsukasa Naganuma, Wataru Sasaki, Naomichi Tanaka, Kazuhiko Kuinose, Kei Matsumoto, Yoshifumi Ikeda, Kazuo Matsumoto, Ritsushi Kato

**Affiliations:** ^1^ Department of Cardiology Saitama Medical University, International Medical Center Hidaka Japan

**Keywords:** ablation skill, atrial fibrillation, CARTONET, pulmonary vein isolation

## Abstract

**Introduction:**

Pulmonary vein isolation (PVI) is a cornerstone of atrial fibrillation (AF) ablation. Unlike balloon‐based or pulsed field ablation, radiofrequency ablation requires precise catheter control and poses a steeper learning curve. This study aimed to compare technical parameters between attending and fellow electrophysiologists (EPs) using data extracted via cloud‐based analysis.

**Methods:**

A total of 194 patients undergoing first‐time AF ablation between April 2023 and September 2024 were analyzed. Procedural metrics, including contact force (CF), inter‐lesion time (ILT), and catheter stability, were compared across six anatomical regions between three attending and five fellow EPs.

**Results:**

Attending EPs achieved significantly shorter ILTs in PVI with moderate‐to‐large effect sizes (Attending vs. Fellows, sec; right PV, 4.8 [4.3–8.4] vs. 9.6 [5.5–15.6], *p* < 0.001, Hedges' *g* = −0.578; left PV, 6.7 [4.5–12.8] vs. 12.7 [6.9–21.7], *p* < 0.001, Hedges' *g* = −0.473), while CF and catheter stability showed smaller differences. In non‐PV regions such as the CTI and roof line, ILT differences were smaller, suggesting early skill acquisition in linear ablation areas by fellows.

**Discussion:**

ILT best reflected operator experience, with shorter ILTs likely contributing to improved lesion continuity and procedural proficiency. Fellows demonstrated comparable CF and catheter stability, potentially aided by steerable sheath use. Mastery of anatomical navigation may be key to advancing proficiency.

**Conclusion:**

Shorter ILTs by attending EPs reflect greater procedural proficiency. Emphasizing anatomical understanding and deliberate catheter manipulation during training may enhance skill development and improve clinical outcomes.

## Graphical abstract

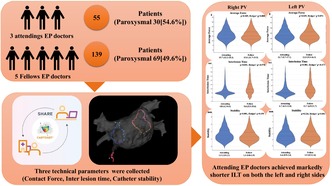

**Abbreviations**
AF: atrial fibrillation
CTI: cavo‐tricuspid isthmus
LPV: left pulmonary vein
PV: pulmonary veins
PVI: pulmonary vein isolation
RPV: right pulmonary vein
SVC: superior vena cava

## 1 Background

Pulmonary veins (PVs) play an important role as a trigger and substrate for atrial fibrillation (AF), and pulmonary vein isolation (PVI) remains a cornerstone strategy in the treatment of AF [1, 2, 3, 4]. Balloon‐based ablation and pulsed field ablation (PFA) allow for rapid isolation of the PVs and have been widely adopted in recent clinical practice [5, 6, 7, 8, 9]. In contrast, radiofrequency (RF) ablation requires point‐by‐point lesion creation around the PV antrum area, necessitating a longer learning curve to achieve procedural proficiency. Incomplete lesion formation may lead to PV reconnections in the chronic phase, resulting in recurrence of AF [10, 11]. Therefore, improving the efficiency of skill acquisition for RF ablation is a key challenge in the training of trainee electrophysiologists (EPs), with the potential to also reduce AF recurrence rates. However, the specific procedural differences between attending EPs and fellow EPs have not been sufficiently investigated. With the recent availability of CARTONET, which enables automated extraction of detailed procedural parameters during catheter ablation [12, 13], we aim to evaluate the technical differences between attending and fellow EPs.

## 2 Methods

### 2.1 Study Patients and Patient Data

This study enrolled 194 patients who underwent their first catheter ablation session for AF using the CARTO 3 system (Biosense Webster Inc., Irvine, CA) between April 2023 and September 2024. This study was performed in accordance with the provisions of the Declaration of Helsinki and local regulations. The research protocol was approved by the hospital's institutional review board (2025‐020).

### 2.2 Ablation Procedure

Procedures were conducted under deep sedation with a target bispectral index (BIS) range of 40–60, utilizing propofol, dexmedetomidine, and pentazocine. Ablations were performed using either the THERMOCOOL SMARTTOUCH SF catheter (STSF; Biosense Webster Inc., Irvine, CA) or the QDOT MICRO catheter (QDOT; Biosense Webster Inc., Irvine, CA). The ablation catheter was manipulated via a steerable sheath (VISIGO, Biosense Webster Inc., Irvine, CA). The ablation protocols were as follows:

After the LA access, a multi‐spline catheter was used to create the LA geometry, which was then utilized to guide the procedure. For the STSF catheter, during PVI and posterior wall isolation, ablation was performed with a power output of 35–40 W, targeting an ablation index (AI) of 450. For lesions in proximity to the esophagus, ablation was applied at 40 W for 7 s or until the esophageal temperature reached 39°C. The cavotricuspid isthmus (CTI) ablation protocol was identical to that used for PVI. Superior vena cava (SVC) isolation was also performed using the same contact force and power settings as for PVI, with a target AI of 350. When the QDOT catheter was used for PVI and posterior wall isolation, two energy delivery modes were employed at the operator's discretion: Q‐mode (50 W, target temperature 47°C, target AI of 450, with automatic termination when the temperature exceeded 55°C) and Q‐mode+ (90 W for 4 s, target temperature 55°C, with automatic termination above 60°C). Ablation near the esophagus was performed exclusively using Q‐mode+. For CTI ablation, power was set at 35 W, with an AI target of 450 or ablation continued for up to 30 s. All SVC isolations were conducted using Q‐mode+.

Real‐time automated display of RF applications (Visitag, Biosense Webster Inc.) was used with predefined settings of catheter stability (2 mm for 3 s) and minimum CF (25% of time > 3 g). All ablation lesions were performed with an interlesion distance of 6 mm or less [14].

### 2.3 Analysis of Procedural Metrics in Radiofrequency Catheter Ablation

Using CARTONET R14, we collected data on the following three technical parameters: contact force (CF), interlesion time (ILT), and catheter stability. CF was calculated as the mean contact force during energy delivery. ILT was defined as the time interval between the end of one application and the start of the subsequent application. Catheter stability was defined as the standard deviation of the catheter tip position, indicating the spatial variability at a given ablation site. Touch‐up ablation sites were excluded from the analysis due to the need for electrical reassessment, and only data from the initial ablation at each site were included. Sites where ablation was initiated following catheter repositioning to a different anatomical location (e.g., from the right PV to the left PV) were excluded from the analysis.

Procedural details, including procedure time, fluoroscopic time, ablation parameters, incidence of first‐pass isolation (FPI), complication details, and AF recurrence, were investigated. Procedure time was defined as the skin‐to‐skin time. AF recurrence was defined as an episode of AF lasting ≥ 30 s and was assessed using Holter electrocardiography and standard 12‐lead electrocardiograms.

The above parameters were compared between three attending EPs (Post graduate year [PGY] ≥ 15, *n* = 2; 15 > PGY ≥ 10, *n* = 1) and five fellow EPs (15 > PGY ≥ 10, *n* = 2; 10 > PGY, *n* = 3) across six anatomical regions: the left and right PVs, roof line, floor line, CTI line, and SVC isolation line. For detailed comparison, the right PVs were classified into four segments (inferior/anterior/roof/posterior) and the left PVs into five segments (inferior/anterior/ridge/roof/posterior).

### 2.4 Statistical Analysis

The statistical analyses were performed using Python (Python 3.11.0) software. Data are expressed as the median with the IQR. The continuous variables were compared using the Mann–Whitney test for the nonparametric data. A chi‐squared test compared the categorical data. Effect sizes were calculated using Hedges' *g* to account for differences in sample sizes between groups. Recurrence of AF was analyzed using Kaplan–Meier analysis. Two‐sided *p*‐values < 0.05 were considered statistically significant.

## 3 Results

### 3.1 Case Characteristics

This study included 194 patients in total, comprising 55 in the Attending group (Attending 1, *n* = 2; Attending 2, *n* = 28; Attending 3, *n* = 25) and 139 in the Fellow group (Fellow 1, *n* = 29; Fellow 2, *n* = 16; Fellow 3, *n* = 30; Fellow 4, *n* = 35; Fellow 5, *n* = 29). Table 1 summarizes the baseline characteristics of the study population. No significant differences were observed in background characteristics between the two groups.

**TABLE 1:** Baseline characteristics of the patients.

	Attending group (*n* = 55)	Fellow group (*n* = 139)	*p*
**Clinical parameters**			
Age	70 (62–76)	71 (63–76)	0.9649
Gender, male, *n* (%)	36 (65.5)	87 (62.6)	0.7082
Height, cm	162 (153–170)	165 (155–170)	0.5377
Body weight, kg	59 (51.7–69.1)	63.4 (53.7–70.3)	0.2571
Paroxysmal AF, *n* (%)	30 (54.6)	69 (49.6)	0.5377
CHADS2 score	1 (0–2)	1 (0–2)	0.4093
Hypertension, *n* (%)	25 (45.6)	48 (34.5)	0.1597
Diabetes mellitus, *n* (%)	7 (12.7)	10 (7.19)	0.2345
CHF, *n* (%)	14 (25.5)	33 (23.7)	0.8024
Stroke, *n* (%)	4 (7.3)	9 (6.5)	0.8424
**Medical therapy**			
Antiarrhythmic drugs, *n* (%)	12 (21.8)	31 (22.3)	0.9417
Oral Anticoagulant, *n* (%)	55 (100)	139 (100)	—
**Laboratory data**			
Creatinine (mg/dL)	0.86 (0.69–1.02)	0.9 (0.76–1.06)	0.2284
NT‐Pro BNP (pg/mL)	651.5 (145.3–1691.5)	582.5 (206.3–1775.8)	0.7861
**Echocardiographic finings**			
LVEF, %	60 (48–66)	58 (49–67)	0.8281
LA diameter, mm	44 (38–48)	43 (37–47)	0.1805

*Note:* Continuous variables are shown as the median (first–third quartile) and categorical variables as the number (%).

Abbreviations: AF, atrial fibrillation; CHF, congestive heart failure; LA, left atrial; LVEF, left ventricular ejection fraction.

### 3.2 Procedure Results

Table 2 shows the procedural results. Procedure and fluoroscopy times were significantly shorter in the Attending group than in the Fellow group. PVI was successfully performed in all cases. CTI ablation, SVC isolation, and posterior wall isolation were performed significantly more frequently in the Attending group. One case in the Attending group who underwent posterior wall isolation developed postoperative gastric hypomotility; however, no statistically significant differences in procedural complications were observed between the Attending and Fellow groups.

**TABLE 2:** Procedure details.

	Attending group (*n* = 55)	Fellow group (*n* = 139)	*p*
**Procedural data**			
Procedure time (min)	74 (60–94)	107 (89–130)	< 0.0001
Fluoroscopy time (min)	9.42 (5.56–14.5)	17.3 (13.3–22.9)	< 0.0001
QDOT catheter, *n* (%)	50 (90.9)	131 (94.2)	0.4024
STSF catheter, *n* (%)	5 (9.1)	8 (5.8)	0.4024
PVI, *n* (%)	55 (100)	139 (100)	—
CTI ablation, *n* (%)	55 (100)	133 (95.7)	0.0435
SVC isolation, *n* (%)	52 (94.5)	42 (30.2)	0.0037
Posterior wall isolation, *n* (%)	12 (21.8)	6 (4.3)	0.0004
**Procedural complications**			
Complications (%)	1 (1.8)	0 (0)	0.1110
Cardiac tamponade, *n* (%)	0 (0)	0 (0)	—
Thromboembolic events, *n* (%)	0 (0)	0 (0)	—
Gastric hypomotility, *n* (%)	1 (1.8)	0 (0)	—
**Procedure details**			
Right PVI			
First‐pass isolation of RPV, *n* (%)	40 (72.7)	114 (82.0)	0.1496
Touch up for Gap‐related site, *n* (%)	1 (1.8)	3 (2.2)	0.8806
Touch up for Carina, *n* (%)	14 (25.5)	22 (15.8)	0.1200
Left PVI			
First‐pass isolation of LPV, *n* (%)	51 (92.7)	117 (84.2)	0.1149
Touch up for Gap‐related site, *n* (%)	0 (0)	3 (2.2)	0.2722
Touch up for Carina, *n* (%)	4 (7.3)	19 (13.7)	0.2142

*Note:* Continuous variables are shown as the median (first–third quartile) and categorical variables as the number (%).

Abbreviations: CTI, cavo‐tricuspid isthmus; LPV, left pulmonary vein; PVI, pulmonary vein isolation; RPV, right pulmonary vein; SVC, superior vena cava.

The procedure time required for each anatomical site was as follows: (Attending vs. Fellows, sec; RPV, 364.3 [315.5–521.4] vs. 727.5 [513.9–898.3], *p* < 0.00001; LPV, 596.3 [383.3–1051.3] vs. 877 [665.1–1299.5], *p* < 0.0001; CTI, 305.0 [180.4–548.9] vs. 372.9 [236.1–972.6], *p* = 0.1279; Roof line, 140.4 [122.2–234.4] vs. 122.9 [52.6–619.7]; Floor Line, 140.4 [122.2–234.4] vs. 122.9 [52.6–619.7], *p* = 0.5848; SVC, 97.6 [49.0–157.3] vs. 223.7 [120.3–318.3], *p* = 0.0003).

In PVI, there were no significant differences between the Attending and Fellow groups in the rate of first‐pass isolation or in the presence of carina conduction due to epicardial connections on either side. Similarly, the frequency of touch‐up ablation along the PVI line, attributed to insufficient initial lesions, did not differ significantly between the two groups for either the left or right side.

**FIGURE 1:**
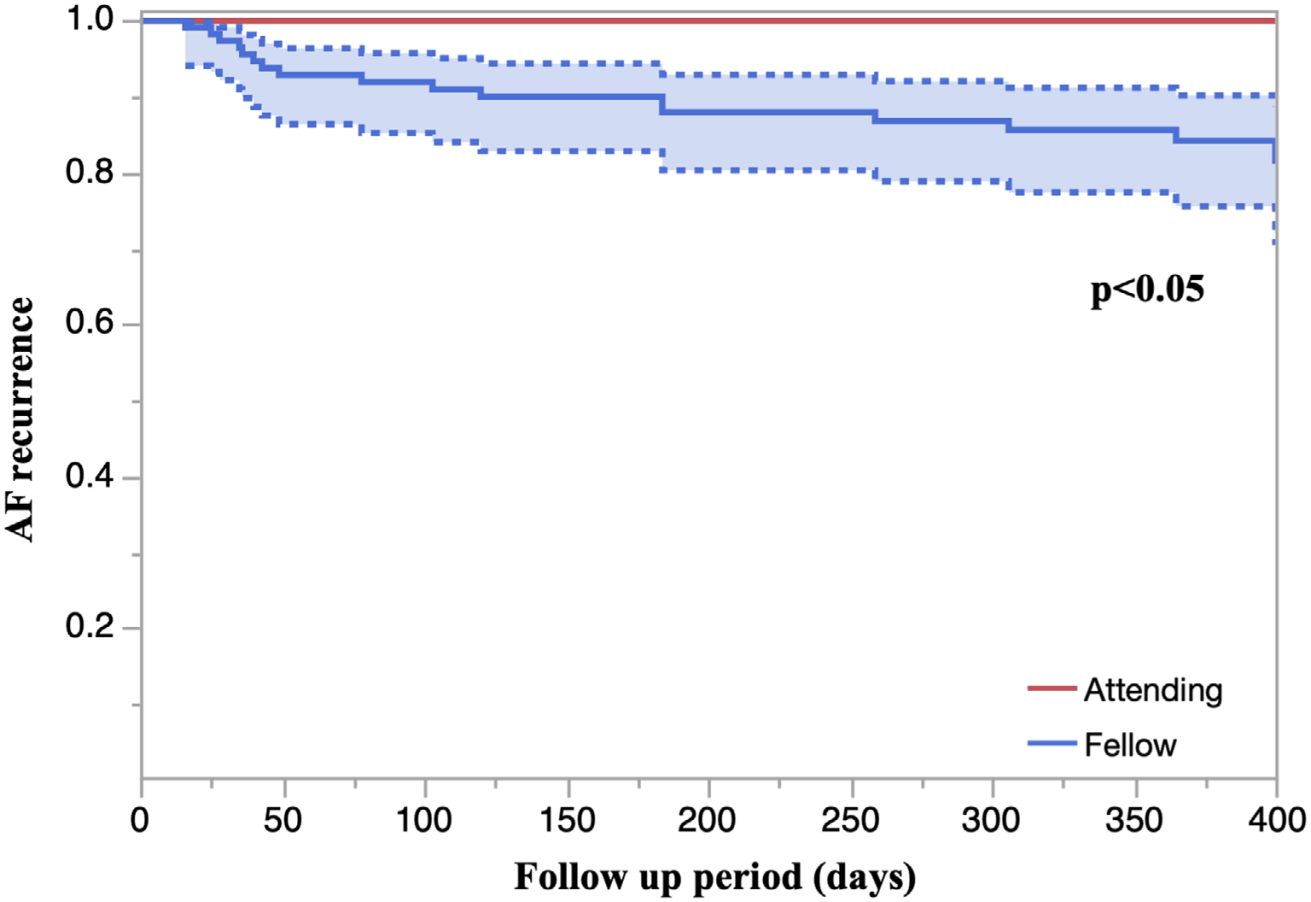
AF recurrence in the two groups. The AF recurrence rate was significantly lower in the attending EP group than in the fellow EP group.

Figure 1 shows AF recurrence in the two groups. The AF recurrence rate was significantly lower in the attending EP group than in the fellow EP group, even though the first‐pass isolation rate did not differ significantly between the two groups.

### 3.3 Comparison of Technical Parameters Between Attending and Fellow Electrophysiologists During Pulmonary Vein Procedures

After excluding touch‐up ablation and cases in which the catheter did not ablate adjacent consecutive points, the remaining 4727 and 11 967 points, respectively, were analyzed (Table S1).

**FIGURE 2:**
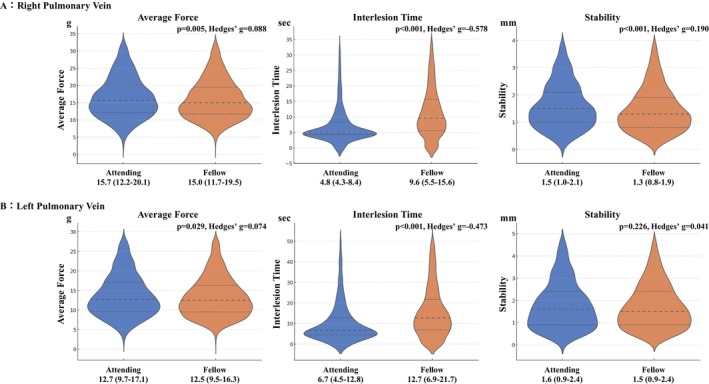
Comparison of ablation parameters for right (A) and left PVs (B) between attending and fellow electrophysiologists. The average CF tended to be significantly higher in attending EPs for both right and left PVs, although the effect sizes were small. ILT was significantly shorter in the attending group on both sides, with moderate effect sizes. Regarding catheter stability, attending EPs showed significantly greater variation in the right PVs, although the effect size was relatively small.

Figure 2 shows the comparison of technical parameters between the attending and fellow EPs. The average force tended to be significantly higher in the attending EPs for both the left and right pulmonary veins, although the effect sizes were small on both sides (Attending vs. Fellows, g; right PV, 15.7 [12.2–20.1] vs. 15.0 [11.7–19.5], *p* = 0.005, Hedges' *g* = 0.088; left PV, 12.7 [9.7–17.1] vs. 12.5 [9.5–16.3], *p* = 0.029, Hedges'*g* = 0.074). For ILT, the attending group showed significantly shorter times on both sides, with moderate effect sizes (Attending vs. Fellows, sec; right PV, 4.8 [4.3–8.4] vs. 9.6 [5.5–15.6], *p* < 0.001, Hedges' *g* = −0.578; left PV, 6.7 [4.5–12.8] vs. 12.7 [6.9–21.7], *p* < 0.001, Hedges' *g* = −0.473). Regarding catheter stability, attending EPs demonstrated significantly greater variation in the catheter tip position in the right PVs, but the effect size was relatively small (Attending vs. Fellows, mm; right PV, 1.5 [1.0–2.1] vs. 1.3 [0.8–1.9], *p* < 0.001, Hedges' *g* = 0.190; left PV, 1.6 [0.9–2.4] vs. 1.5 [0.9–2.4], *p* = 0.226, Hedges' *g* = 0.041).

**FIGURE 3:**
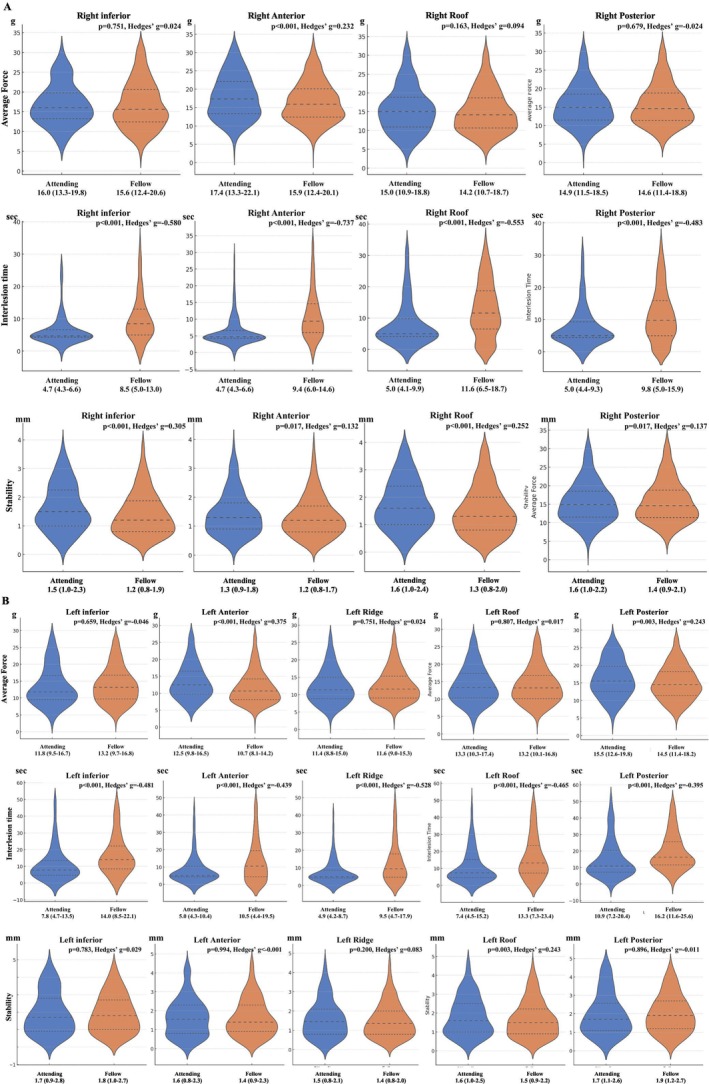
Site‐specific comparison of ablation parameters. (A) The comparison of parameters at each ablation site in the right PVs. Attending EPs tended to apply a higher average CF at the right anterior region only, but the effect sizes were small across all sites. No significant differences in CF were observed at other locations. However, ILT was shorter at all sites in the attending group, and catheter stability was consistently lower. Notably, the effect sizes were consistently larger for ILT than for catheter stability. (B) The comparison at each site in the left PVs. Attending EPs applied a higher average CF particularly at the left anterior and posterior regions. ILT was shorter at all sites in the attending group. Catheter stability had significantly greater variation at the left roof in the attending group. Across all sites, the largest effect sizes were seen for ILT.

Figure 3A shows the comparison of these parameters at each ablation site in the right PVs. For average force, attending EPs tended to apply higher force only at the right anterior area, but the effect sizes were small at all sites. At other sites, no significant differences in CF were observed between attending and fellow EPs. However, ILT was shorter in all sites for the attending doctors, and catheter stability had greater variation across all sites in the attending group. Nevertheless, the effect sizes were consistently larger for ILT than for catheter stability.

Figure 3B shows the comparison of these parameters at each ablation site in the left PVs. For average force, attending EPs tended to apply higher force at the left anterior and left posterior areas. ILT was shorter at all sites in the attending group. Catheter stability had significantly greater variation in the attending EPs at the left roof. However, effect sizes were greatest for ILT across all sites.

### 3.4 Comparison of Technical Parameters Between Attending and Fellow Electrophysiologists in Non‐Pulmonary Vein Areas

**FIGURE 4:**
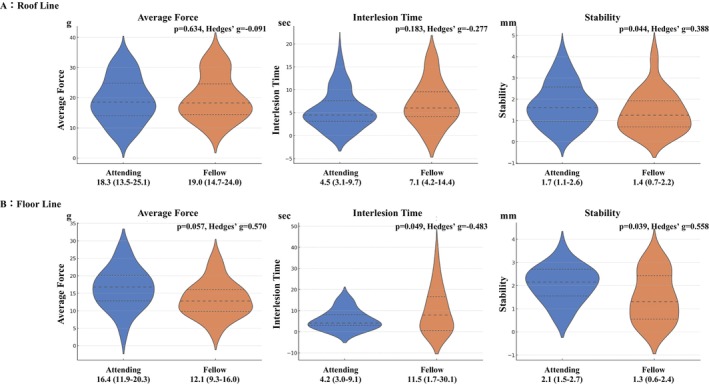
Comparison of ablation parameters during posterior wall isolation. For the roof line, catheter stability had significantly greater variation in attending EPs. At the floor line, ILT was significantly shorter and catheter stability had significantly greater variation in the attending group; however, the effect size was greater for catheter stability.

**FIGURE 5:**
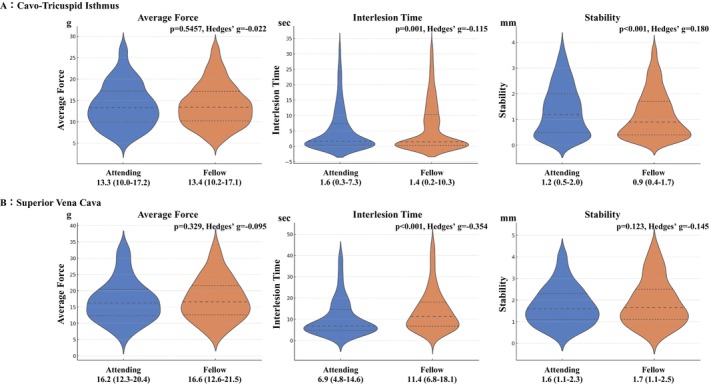
Comparison of ablation parameters in the right atrium. During CTI ablation, ILT was significantly shorter, and catheter stability had greater variation in the attending group, though both parameters had small effect sizes. In SVC isolation, ILT was also significantly shorter in attending EPs.

Figures 4 and 5 present the comparison of parameters at sites other than PVI. For the roof line, catheter stability had greater variation in the attending EPs. At the floor line, ILT was significantly shorter and catheter stability had greater variation in the attending EPs; however, the effect size was greater for catheter stability. During ablation of the CTI line, ILT was significantly shorter and catheter stability had greater variation in the attending EPs; however, the effect sizes for both parameters were small. In the SVC isolation, ILT was also significantly shorter in the attending EPs.

## 4 Discussion

### 4.1 Major Findings

The major findings of our study were as follows:

1. In PVI, attending EPs achieved markedly shorter ILT on both the left and right sides, accompanied by large effect sizes. While differences in catheter stability were modest, attending EPs generally exhibited greater variation indices compared to fellows. CF differences between groups were minimal, reflected by small effect sizes across all pulmonary veins.
2. When analyzing individual segments of all PVs, ILT also consistently emerged as the parameter with the largest effect size.
3. In CTI ablation, ILT and catheter stability differed only slightly between two groups, with small corresponding effect sizes. During SVC isolation, attending EPs again demonstrated shorter ILT.

### 4.2 Regarding Differences in ILT Across Ablation Sites

ILT was shorter across all segments for attending EPs. However, the effect size was small in regions such as the CTI and roof line. These areas are typically treated with linear ablation and require relatively simple three‐dimensional catheter manipulation. As such, fellows may achieve early proficiency in catheter control in these anatomically straightforward regions. Furthermore, on the roof line, respiratory movement may affect catheter stability and CF, which could explain the delay in initiating ablation to the next point, even among attending electrophysiologists. In contrast, at sites requiring more complex spatial manipulation—such as the PVs or SVC—attending EPs demonstrated significantly shorter ILTs. This suggests that advanced anatomical understanding and the ability to perform precise three‐dimensional catheter movements are critical skills that require further development during fellowship training.

### 4.3 The Importance of ILT in Catheter Ablation

In catheter ablation, lesion formation is primarily influenced by CF, power output, and application duration—parameters that reflect the characteristics of individual ablation points [15, 16, 17].

However, PVI requires the creation of a continuous lesion set, and thus evaluating lesion continuity is equally essential. In our cohort, only 0.9%–2.0% of cases were excluded from the analysis because of skip lesions, and lesion placement was generally contiguous except in areas adjacent to the esophagus. Moreover, ILT consistently showed the largest effect size among the evaluated parameters, supporting its role as a particularly relevant surrogate marker of lesion continuity. Shorter ILTs have been reported to be associated with greater inter‐lesion depth and improved transmurality between adjacent ablation points [18]. Although the rate of first‐pass isolation did not significantly differ between the two groups, the AF recurrence rate was lower in the attending EP group than in the fellow EP group. This finding suggests that shorter ILT may contribute to better lesion continuity and more durable PVI. Therefore, minimizing ILT through careful catheter manipulation during lesion delivery may improve long‐term procedural outcomes.

Although the rate of first‐pass isolation did not significantly differ between the two groups, the AF recurrence rate was lower in the attending EP group than in the fellow EP group. This finding suggests that shorter ILT may contribute to better lesion continuity and more durable PVI. Therefore, minimizing ILT through careful catheter manipulation during lesion delivery may improve long‐term procedural outcomes.

### 4.4 Technical Skill Development in AF Ablation

Although few studies have objectively evaluated ablation techniques in AF procedures, one prior report indicated that procedural improvements among first‐ or second‐year fellows may not significantly influence lesion‐level outcomes [19]. In contrast to these earlier investigations, our study directly compared technical parameters between fellows and experienced attending EPs, thereby capturing differences that may reflect long‐term procedural refinement.

Our findings indicate that CF did not differ significantly between fellows and attendings in most anatomical locations. Although catheter stability had significantly greater variation in the attending group, the effect size was small, suggesting that fellows were able to achieve a comparable level of stability control even at early stages of training. In this study, a steerable sheath was utilized in all procedures, which may have contributed to the early acquisition of catheter stability among fellows. The consistent use of steerable sheaths may play a supportive role in minimizing differences in stability between operators with varying levels of experience. This fact suggests that fellows are able to acquire adequate CF control early in their training. In contrast, ILT was notably shorter among attending EPs in many regions, with large effect sizes observed. This disparity may reflect a higher level of anatomical awareness among attendings, allowing for faster and more precise catheter repositioning between lesions.

These findings imply that mastering the anatomy is a key factor in bridging the skill gap between fellows and experienced operators. Importantly, such anatomical familiarity need not be developed solely through direct clinical experience. Our study suggests the possibility that simulation‐based training using anatomical models may offer a valuable adjunctive pathway for improving catheter navigation skills and procedural efficiency. This highlights the potential role of structured simulation programs in enhancing the technical proficiency of trainees in AF ablation.

## 5 Limitations

Our study has several limitations. Firstly, although there were no significant differences in baseline patient characteristics between cases performed by attending and fellow EPs, attending EPs more frequently performed additional procedures such as posterior wall isolation and SVC isolation. Due to these variations in adjunctive ablation strategies beyond PVI, it was not feasible to accurately assess total procedural duration or compare recurrence rates across groups. Secondly, the frequency of posterior wall isolation differed between two groups, making it difficult to evaluate technical differences in posterior wall ablation. Thirdly, due to the differing number of cases performed within the Attending and Fellow groups, direct comparisons between individual operators were not feasible. During the study period, the fellows also performed ablation using other 3D mapping systems. Therefore, it was not possible to evaluate the learning curve based solely on procedures performed with the CARTO system in this study. Finally, this was a single‐center study. As ablation techniques and procedural protocols may vary across institutions, future investigations involving multiple centers are necessary to validate the generalizability of our findings.

## 6 Conclusion

Attending EPs performed ablation with shorter ILT, which likely contributed to more efficient procedures and reduced overall ablation time. For fellows, enhancing anatomical understanding and achieving shorter ILTs during ablation may be key components of training that facilitate progression toward the technical proficiency of experienced operators.

## Author Contributions

H.M. provided the study concept and design and drafted the manuscript; D.K., M.N., K.M., T.N., W.S., N.T., K.M., K.K., K.M., and H.M. contributed to the data analysis and interpretation. Y.I., K.M., and R.K. reviewed and revised the manuscript draft.

## Funding

The authors have nothing to report.

## Disclosure

H.M. received lecture fees from Biosense Webster Japan and Boston Scientific Japan. Our department received grant support from Boston Scientific Japan and Abbott Medical Japan. No AI tools were used for data analysis, study design, or interpretation of results.

## Ethics Statement

The study protocol was approved by the hospital's institutional review board (IRB number: 2025‐020).

## Conflicts of Interest

The authors declare no conflicts of interest.

## Supporting information


**Table S1a:** Detailed number of ablation Points (Right PV).
**Table S1b:** Detailed number of ablation Points (Left PV).
**Table S1d:** Detailed number of ablation Points (Floor Line).
**Table S1e:** Detailed number of ablation Points (CTI Line).
**Table S1f:** Detailed number of ablation Points (SVC).

## Data Availability

The data that support the findings of this study are available on request from the corresponding author. The data are not publicly available due to privacy or ethical restrictions.
