# Supplementary material for: Interlesion Time as a Key Metric of Procedural Proficiency in Atrial Fibrillation Ablation: From Fellow to Attending
Source: J Arrhythm. 2026 May 14;42(3):e70360. doi: 10.1002/joa3.70360 (PMC13176641; doi:10.1002/joa3.70360)
Supplement: Supplementary file 1 — Table S1a: Detailed number of ablation Points (Right PV). Table S1b: Detailed number of ablation Points (Left PV). Table S1d: Detailed number of ablation Points (Floor Line). Table S1e: Detailed number of ablation Points (CTI Line). Table S1f: Detailed number of ablation Points (SVC). [file JOA3-42-e70360-s001.docx]

**Table S1a. Detailed number of ablation Points (Right PV)**

|  | Attending (n=1669) | Fellow (n=4504) |
| --- | --- | --- |
| Excluded Point | | |
| Touch Up Site, n (%) | 45 (2.7) | 88 (2.0) |
| Non-adjacent ablation points, n (%) | 15 (0.9) | 39 (0.9) |
| Ablation Setting | | |
| QMODE, n (%) | 473 (29.4) | 2405 (54.9) |
| QMODE+, n (%) | 1032 (64.1) | 1709 (39.0) |
| LEGACY, n (%) | 104 (6.5) | 263 (6.0) |
| Analyzed Ablation Points | | |
| Right Anterior, n (%) | 525 (32.6) | 1404 (32.1) |
| Right Inferior, n (%) | 251 (15.6) | 730 (16.7) |
| Right Posterior, n (%) | 462 (28.7) | 1292 (29.5) |
| Right Roof, n (%) | 371 (23.1) | 951 (21.7) |

**Table S1b. Detailed number of ablation Points (Left PV)**

|  | Attending (n=1473) | Fellow (n=4207) |
| --- | --- | --- |
| Excluded Point | | |
| Touch Up Site, n (%) | 26 (1.8) | 61 (1.4) |
| Non-adjacent ablation points, n (%) | 28 (2.0) | 45 (1.1) |
| Ablation Setting | | |
| QMODE, n (%) | 316 (22.3) | 1543 (37.6) |
| QMODE+, n (%) | 999 (70.4) | 2308 (56.3) |
| LEGACY, n (%) | 104 (7.3) | 250 (6.1) |
| Analyzed Ablation Points | | |
| Left Anterior, n (%) | 252 (17.8) | 745 (18.2) |
| Left Inferior, n (%) | 182 (12.8) | 604 (14.7) |
| Left Posterior, n (%) | 267 (18.8) | 874 (21.3) |
| Left Ridge, n (%) | 391 (27.6) | 1000 (24.4) |
| Left Roof, n (%) | 327 (23.0) | 878 (21.4) |

**Table S1c. Detailed number of ablation Points (Roof Line)**

|  | Attending (n=164) | Fellow (n=60) |
| --- | --- | --- |
| Excluded Point | | |
| Touch Up Site, n (%) | 34 (20.7) | 7 (11.7) |
| Non-adjacent ablation points, n(%) | 7 (4.3) | 2 (3.3) |
| Ablation Setting | | |
| QMODE, n (%) | 60 (48.8) | 31 (60.8) |
| QMODE+, n (%) | 63 (51.2) | 20 (39.2) |
| LEGACY, n (%) | 0 (0) | 0 (0) |
| Analyzed Ablation Points | | |
| Roof Line, n (%) | 123 (100) | 51 (100) |

**Table S1d. Detailed number of ablation Points (Floor Line)**

|  | Attending (n=191) | Fellow (n=87) |
| --- | --- | --- |
| Excluded Point | | |
| Touch Up Site, n (%) | 61 (31.9) | 2 (2.3) |
| Non-adjacent ablation points, n (%) | 8 (4.2) | 4 (4.6) |
| Ablation Setting | | |
| QMODE, n (%) | 24 (19.7) | 44 (54.3) |
| QMODE+, n (%) | 98 (80.3) | 37 (45.7) |
| LEGACY, n (%) | 0 (0) | 0 (0) |
| Analyzed Ablation Points | | |
| Floor Line, n (%) | 122 (100) | 81 (100) |

**Table S1e. Detailed number of ablation Points (CTI Line)**

|  | Attending (n=1249) | Fellow (n=3028) |
| --- | --- | --- |
| Excluded Point | | |
| Touch Up Site, n (%) | 5 (0.4) | 58 (1.9) |
| Non-adjacent ablation points, n (%) | 8 (0.6) | 9 (0.3) |
| Ablation Setting | | |
| QMODE, n (%) | 1100 (89.0) | 2787 (94.1) |
| QMODE+, n (%) | 0 (0) | 2 (0.1) |
| LEGACY, n (%) | 136 (11.0) | 172 (5.8) |
| Analyzed Ablation Points | | |
| CTI Line, n (%) | 1236 (100) | 2961(100) |

**Supplementary Table 1f. Detailed number of ablation Points (SVC)**

|  | Attending (n=222) | Fellow (n=396) |
| --- | --- | --- |
| Excluded Point | | |
| Touch Up Site, n (%) | 0 (0) | 0 (0) |
| Non-adjacent ablation points, n (%) | 4 | 0 (0) |
| Ablation Setting | | |
| QMODE, n (%) | 0 (0) | 2 (0.5) |
| QMODE+, n (%) | 207 (95.0) | 387 (97.7) |
| LEGACY, n (%) | 11 (5.0) | 7 (1.8) |
| Analyzed Ablation Points | | |
| SVC, n (%) | 218 (100) | 396 (100) |
